# Facile preparation of porous biomass charcoal from peanut shell as adsorbent

**DOI:** 10.1038/s41598-020-72721-0

**Published:** 2020-09-28

**Authors:** Suzhen Bai, Tiantian Wang, Zhengshan Tian, Kesheng Cao, Jitao Li

**Affiliations:** 1 School of Chemistry and Environmental Engineering, Pingdingshan University, Weilai Road, Pingdingshan, 467000 People’s Republic of China; 2 School of Physics and Telecommunications Engineering, Zhoukou Normal University, Wenchang Dong Road, Zhoukou, 466001 People’s Republic of China

**Keywords:** Materials science, Nanoscience and technology

## Abstract

Activated carbons derived from biomass have been proved to be one of the most promising adsorbents due to their abundance, low cost, reproducibility and environmental friendliness. In this study, a simple, facile and effective pyrolysis method was demonstrated to prepare hierarchical porous biomass charcoal by using peanut shells as precursor without chemical activation in an electric muffle furnace. The obtained products hold porous structure and abundant oxygen-containing functional groups, which were mainly due to in-built template of the structure of peanut shell and the preparation process without nitrogen protection, respectively. Interestingly, the obtained biomass charcoal sample with excellent adsorptive property quickly removed Pb^2+^ (100 mg/L) and methylene blue (50 mg/L) from water with removal efficiency of 96.5% and 97.1%, and removal capacity of 48 mg/g and 24 mg/g, respectively. The synthetic process was simple and economical, and it could be used as a beneficial reference in the recycling of biomass waste.

## Introduction

With the awareness of sustainable use of natural resources, the recovery of biomass and biomass waste has been received increasing attention^1–4^. Moreover, abundant and renewable biomass waste has been proved to be promising precursor of carbonaceous materials with lots of emergent applications^5–7^. Because of high density of oxygenated functional groups and low degree of condensation, the biomass waste can be tailored to produce carbon-based materials with desired characteristics for different functional applications. Especially activated carbons produced from biomass waste have been widely used in many fields, such as adsorbent^8,9^, supercapacitor^10–12^, oil–water separation^13^, resource recovery^14^ and so on.

On the other hand, peanut shells are as one of important agricultural residues all over the world, only few of them can be used as a stock feed and building materials in rural areas, most of them are basically not being used and would be discarded. The annual output of raw peanut is huge worldwide, especially the annual output of China is over 16.5 million tons and there are tons of discarded peanut shells every year in China and all over the world^15,16^. From the perspective of sustainable development, peanut shells should not be as a waste material and not be discarded due to their abundance, low cost, reproducibility and environmental friendliness. Therefore, the recycling of peanut shells is of great concern and lots of effective approaches are needed to solve such issues.

In the past years, numerous methods have been reported for the synthesis of activated carbon derived from peanut shells for different applications^8,10,13,16^. For example, Dey et al. reported a process for the production of few-layer graphene from peanut shell for high-performance supercapacitor^17^. In the synthesis of carbon nanomaterials, the peanut shell precursor powder was firstly pyrolyzed in a tubular furnace at 800 °C for 2 h under an argon atmosphere to get activated carbon materials. Recently, the utilization of peanut shell for the adsorptive removal of pollutants has been received immense attention for the purpose of water treatment^8,13,16^. For example, Islam et al. reported the synthesis of a low-cost and high-capacity adsorbent by refluxing the peanut shell in concentrated H_2_SO_4_^18^. The obtained adsorbent could effectively remove methylene blue and tetracycline from water. Sharma and his collaborators prepared a low-cost activated carbon from peanut shells, using H_3_PO_4_ followed by pyrolysis at 650 °C under nitrogen environment, and used as an adsorbent to remove Acid Yellow 36 dye from waste water^19^. In this regard, the utilization of peanut shell to prepare biomass charcoal as an efficient adsorbent for water treatment could provide two benefits. Firstly, it could be a useful application of peanut shell. Secondly, its utilization in water treatment may provide the access of clean water for the people in poor areas.

Although, there are lots of reports for the utilization of peanut shell in the adsorptive removal of organic dyes, heavy metal ion and antibiotics from waste water. However, many preparation processes of these activated carbons were tedious, multiple-step and not green. For example, KOH, H_3_PO_4_, H_2_SO_4_, FeCl_3_, ZnCl_2_ and so on were used as activating agents, and these chemicals would be cleaned out in the subsequent processes, which would bring some environmental pollution. Therefore, a simple, green and scalable route for the development of hierarchical porous biomass charcoal from peanut shells without chemical activating reagents is in urgent need.

In this paper, a simple, green and scalable method was explored to fabricate hierarchical porous biomass charcoal with abundant oxygen-containing functional groups by using peanut shells as the precursors. The biomass charcoal samples were obtained without activating agents and protective gases through a high-temperature pyrolysis process in an electric muffle furnace. Due to large surface area, porous structure and oxygen-containing functional groups, the obtained samples possess perfect adsorptive property to quickly remove Pb^2+^ and methylene blue (MB) from waste water.

## Experimental section

### Materials

The main raw materials, peanut shells, were obtained from Pingdingshan agricultural products market of Henan Province in China. Lead nitrate (Pb(NO_3_)_2_) and methylene blue (MB) were purchased from Aladdin Reagent Co., Ltd. The chemicals used in our experiments were of analytical grade and used without any purification. Deionized water made in our lab was used in this experiment.

### Methods

The peanut shells were collected and used as a source of carbon to prepare biomass charcoal through a high-temperature pyrolysis process. In a typical procedure, the preparation details were described as follows.

Firstly, the peanut shells were washed thoroughly with tap water several times, then rinsed with deionized water under ultrasonic treatment to remove stone, dust, dirt and other impurities, finally dried in the oven of 60 °C for 10 h.

Secondly, the above dried peanut shells were ground into powders and then sieved to remove larger broken pieces through a sieve of 0.2 mm.

Thirdly, appropriate peanut shell powders were placed into a series of porcelain crucibles and covered with lids in an electric muffle furnace without nitrogen protection, then heated to 300 °C, 400 °C, 500 °C, 600 °C, 700 °C, 800 °C, 900 °C and 1000 °C, respectively. The heating rate of the muffle furnace was set at 20 °C/min. All the above reactions of respective temperatures were maintained for 6 h, respectively.

Finally, a series of products were obtained after natural cooling. Based on different reaction temperatures (from 300 °C to 400 °C, 500 °C, 600 °C, 700 °C, 800 °C, 900 °C and 1000 °C), for comparison, these obtained products were marked as Sample 300, Sample 400, Sample 500, Sample 600, Sample 700, Sample 800, Sample 900 and Sample 1000, respectively.

### Characterizations

The morphology and microstructure of the biomass charcoal samples were characterized by a scanning electron microscope (SEM, Hitachi S-4800) and a X-ray diffractometer (XRD, Bruker D8 Discover) with Cu Ka radiation (1.5406 Å). Fourier transform infrared spectrum (FTIR, Nicolet5700), Raman spectrum with an excitation laser of 514.5 nm (LabRAM HR800) and X-ray photoelectron spectroscopy (XPS, ESCALAB 250Xi) were used to analyze the components and the formation process of biomass charcoal samples.

### Adsorption experiments

The Sample 1000 was typically employed as adsorbent to remove Pb^2+^ (100 mg/L) from water. Aqueous solution of Pb^2+^ was prepared by dissolving the corresponding lead nitrate in deionized water to arrive at a concentration of 100 mg/L. Adsorption tests were carried out using 100 mg adsorbent in 50 mL aqueous solution. Batch experiments of adsorption were carried out in glass beakers with stirring at 200 rpm at room temperature. At given time intervals, 5 mL of the mixture solution was retrieved from the solution and centrifuged. The concentration of Pb^2+^ in the supernatant was determined by atomic absorption spectrophotometry (Shimadzu AA-6880)^20,21^.

On the other hand, MB was chosen as the model compound to evaluate the adsorptive effect of Sample 1000 on organic pollutants. The general experimental process was described as follows: 100 mg of Sample 1000 was added to 50 mL MB solution with an initial concentration of 50 mg/L, followed by stirring at 200 rpm at room temperature. At appropriate time intervals, the adsorbent of Sample 1000 was separated from the suspension via filtration and the concentration of residual MB in the supernatant solution was calculated by Beer’s law based on the absorption peak at 662 nm for MB by using a UV–Vis spectrophotometer (Shimadzu UV-2450)^22,23^.

## Results and discussion

### Structural analysis

**Figure 1:**
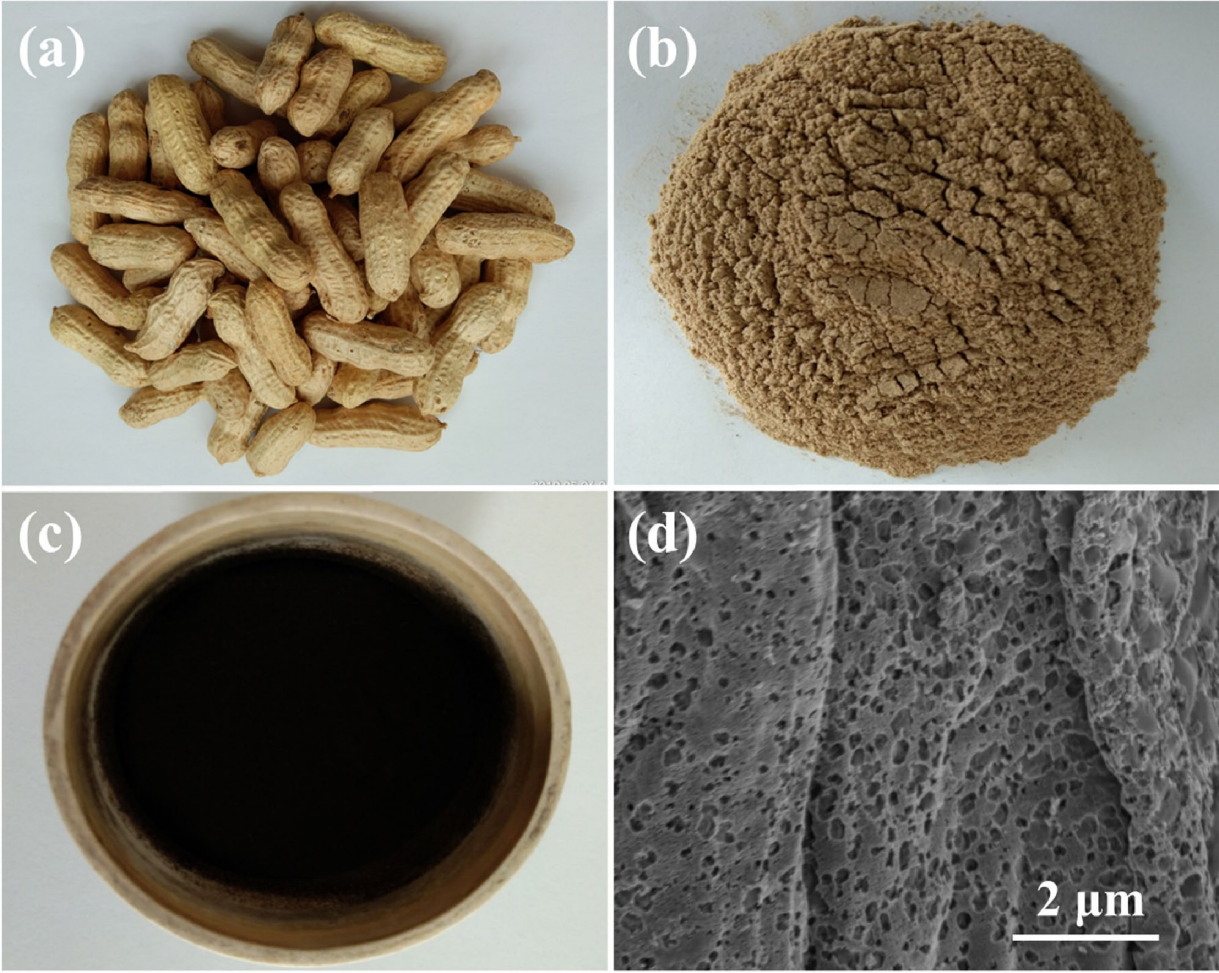
Optical photographs of (**a**) peanut shells, (**b**) peanut shell powders and (**c**) biomass charcoal of Sample 1000 in a porcelain crucible. (**d**) SEM image of Sample 1000.

The optical photographs of peanut shells, peanut shell powders and Sample 1000 were shown in Fig. 1. It can be seen that the colors of the peanut shells and peanut shell powder are pale yellow (Fig. 1a,b), while the color of the biomass charcoal of Sample 1000 served in porcelain crucible is jet black (Fig. 1c). SEM image in Fig. 1d shows that Sample 1000 has a thin sheet with hierarchical porous structure.

**Figure 2:**
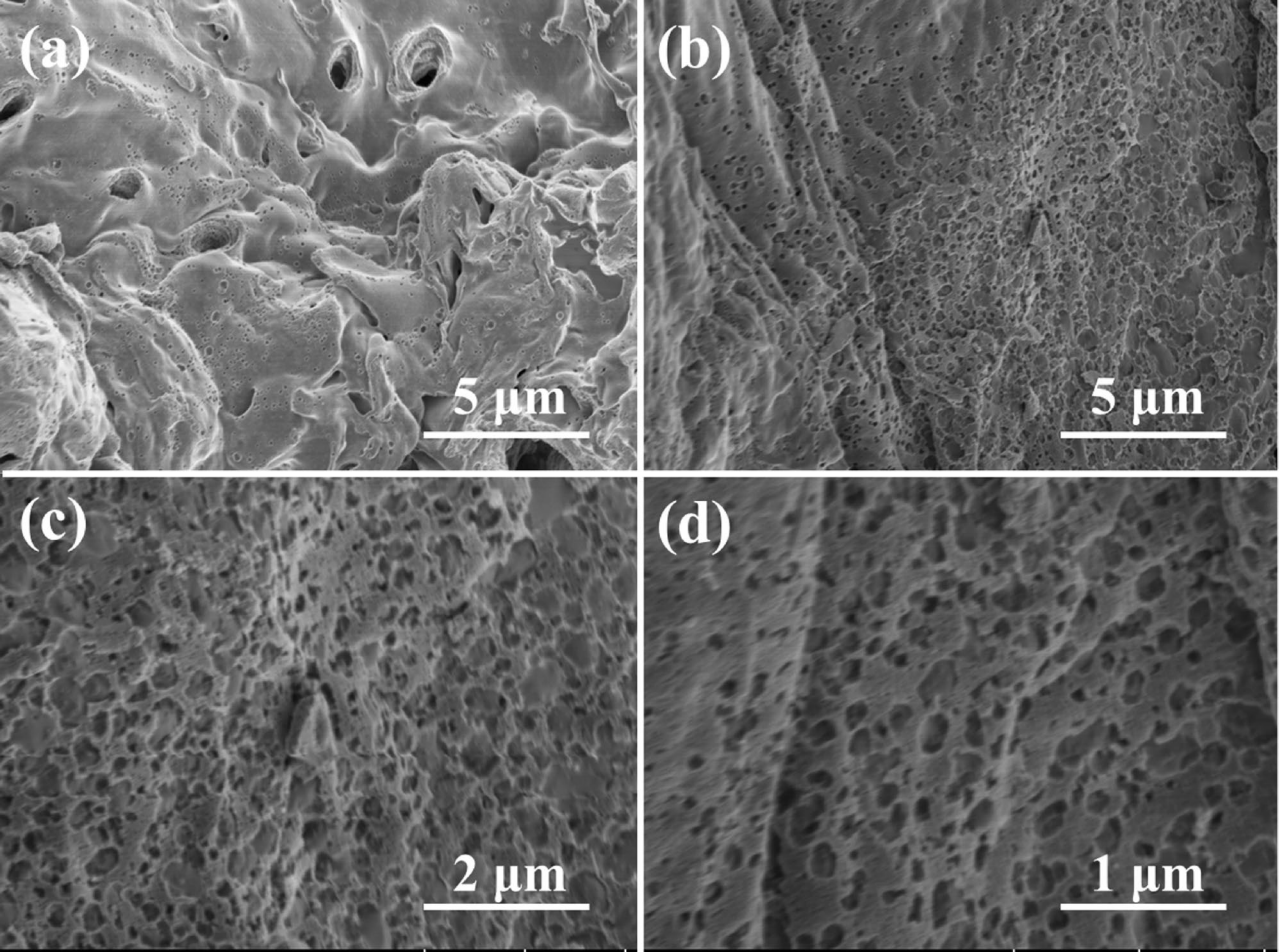
(**a**) SEM image of peanut shell powders and (**b–d**) SEM images of Sample 1000 with different magnifications.

In our experiments, a scale preparation of biomass charcoal with porous structure can be readily achieved. To reveal their microstructure, SEM images of peanut shell powders and Sample 1000 were typically carried out and shown in Fig. 2. It can be seen from SEM images that peanut shell powders hold an irregular large sheet structure (Fig. 2a). While the biomass charcoal of Sample 1000 possesses a good morphology (Fig. 2b–d) with a loose and interconnected macroporous structure (100–500 nm), which is apparently different from a relative close-packed structure of peanut shell powders (Fig. 2a).

**Figure 3:**
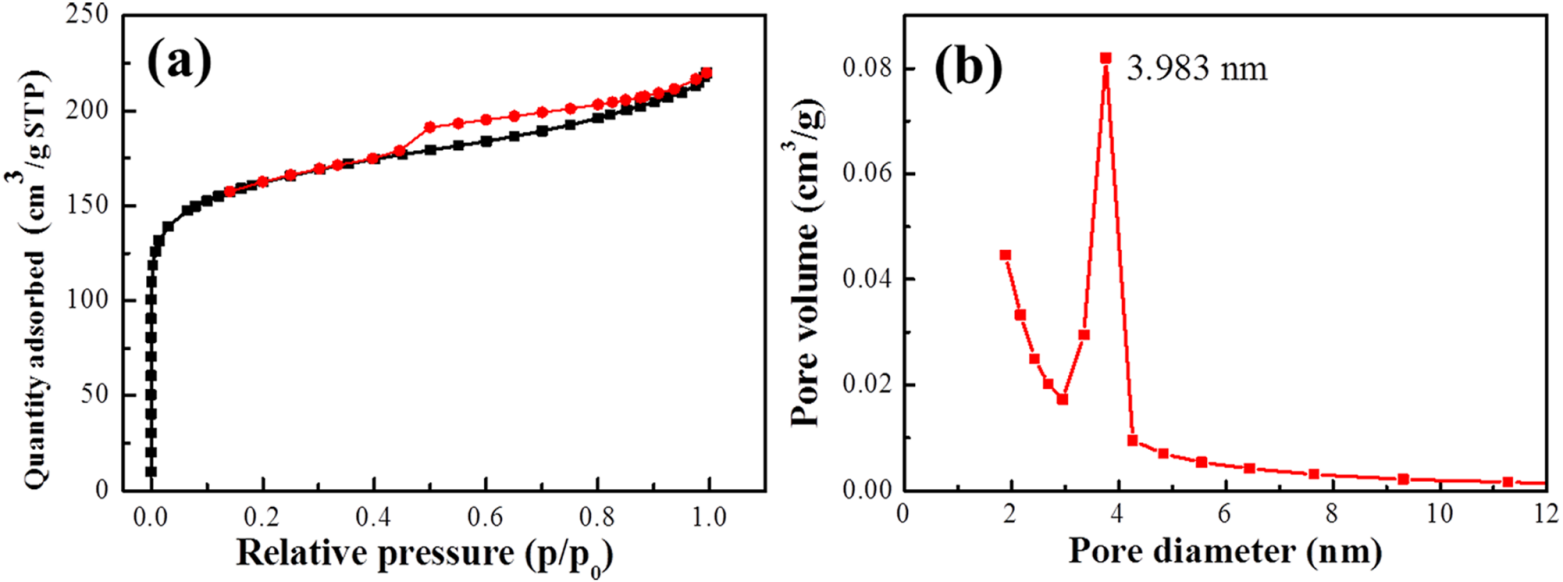
(**a**) N_2_ adsorption–desorption isotherms and (**b**) Pore-size distribution of Sample 1000.

To clarify the average pore size and other porosity parameter, N_2_ adsorption–desorption at 77 K was carried out with surface area and pore size analyzer (Micromeritics, Gemini analyzer). The specific surface area was calculated by the conventional Brunauer–Emmett–Teller (BET) method and the pore-size distribution was determined by the Barrett–Joyner–Halenda (BJH) model, as shown in Fig. 3.

The isotherm in Fig. 3a exhibited a typical N_2_ adsorption–desorption isotherm of type IV with a sharp rise at low relative pressure (P/P_0_ < 0.10) and a B-type hysteresis loop at high relative pressure (P/P_0_ > 0.45), suggesting the existence of micropores, mesopores and external surface area in the porous materials^24–26^. The BET surface area of Sample 1000 was 554. 528 m^2^/g, including micropore area of 340.696 m^2^/g and external surface area of 213.832 m^2^/g. The pore volume and micropore volume of Sample 1000 were 0.330 cm^3^/g and 0.157 cm^3^/g, respectively. BJH adsorption average pore diameter was 3.983 nm (Fig. 3b). According to the International Union of Pure and Applied Chemistry (IUPAC) classification, porous materials are classified into three groups: micropore (< 2 nm), mesopore (2–50 nm) and macropore (> 50 nm). Thus, the biomass charcoal sample has a hierarchical porous structure containing micropores, mesopores and macropores.

**Figure 4:**
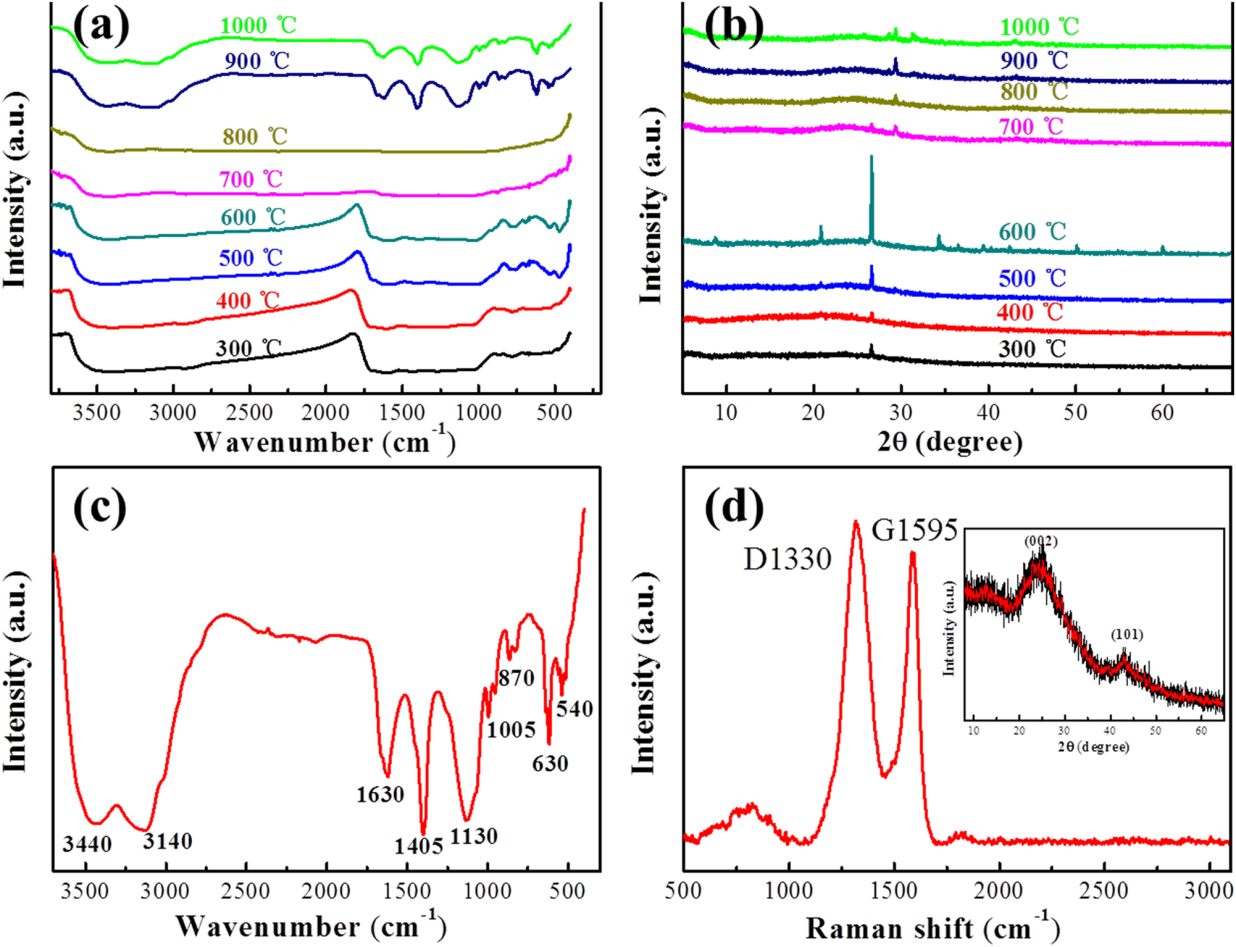
(**a**) FTIR spectra and (**b**) XRD patterns of the biomass charcoal samples (Sample 300 to Sample 1000); (**c**) FTIR spectrum and (**d**) Raman spectrum of Sample 1000, the inset is XRD patterns of Sample 1000.

To further explore the formation process of biomass charcoal with hierarchical porous structure, it is generally considered that the pyrolysis temperature is one of the most important influence factors^27^, thus control experiments of different temperatures (from 300 °C to 400 °C, 500 °C, 600 °C, 700 °C, 800 °C, 900 °C and 1000 °C) were performed at a constant heating rate of 20 °C/min under the same experimental conditions. Seen from the FTIR measurements of the samples in Fig. 4, their absorption peaks have significant differences before and after 700° C, that is, the absorption peaks gradually appear changes with a stabilizing tendency from Sample 300 to Sample 600, and then the absorption peaks gradually appear another stabilizing tendency from Sample 700 to Sample 1000 (Fig. 4a). The same changing tendency can be also found in the XRD patterns, as shown in Fig. 4b.

Therefore, it is reasoned that hierarchical porous biomass charcoal can be formally obtained at 700 °C after the reactions from 300 to 700 °C, then the sample structures can be gradually optimized from 700 to 1000 °C. The FTIR spectra revealed that original oxygen-containing functional groups of peanut shell powder were slowly removed from 300 to 700 °C, while newly formed oxygen-containing functional groups were gradually optimized from 700 to 1000 °C due to the pyrolysis process without nitrogen protection.

To explore the functional groups of the biomass charcoal, FTIR and Raman spectra of Sample 1000 were typically analyzed. The FTIR spectra (Fig. 4c) have proved the presence of different functional groups^28,29^. such as O–H stretching vibration around 3440 cm^−1^, C–H stretching vibration around 3140 cm^−1^ and 1405 cm^−1^, C=C stretching vibration around 1630 cm^−1^, C–OH stretching vibration at 1130 cm^−1^ and C–O stretching mode at 1005 cm^−1^.

Raman spectrum of Sample 1000 was shown in Fig. 4d. It can be seen that D peak (~ 1345 cm^−1^) and G peak (~ 1593 cm^−1^) are presented in the Sample 1000, and the intensity ratio of the D band (~ 1345 cm^−1^) to the G band (~ 1593 cm^−1^) (*I*_D_*/I*_G_) is 1.10, very similar to that of graphene oxide reported by the literature^28,29^. Raman spectrum reveals that the biomass charcoal of Sample 1000 has lots of defects, which maybe be caused by the internal structure of the peanut shell, the high temperature pyrolysis of 1000 °C and the subsequent process of natural cooling without the protection of nitrogen. XRD patterns of Sample 1000 display two broad and weak peaks at about 24° and 43° of (002) and (101) planes of hexagonal graphite, respectively, suggesting the presence of amorphous carbons in the biomass charcoal sample (the inset of Fig. 4d) ^24^.

**Figure 5:**
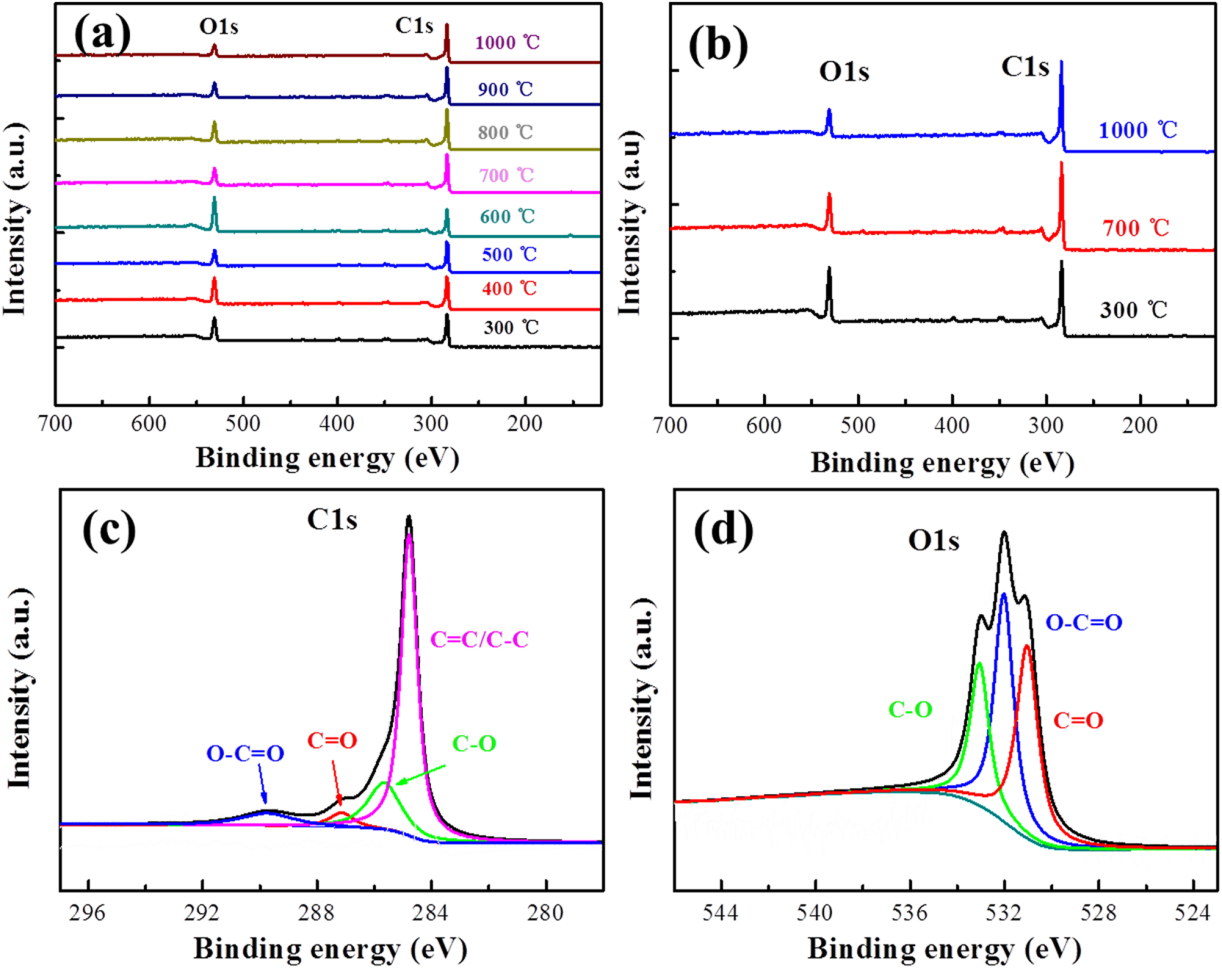
(**a**) Survey spectra of biomass charcoal samples (Sample 300 to Sample 1000), (**b**) Survey spectra of Sample 300, Sample 700 and Sample 1000; (**c**,**d**) C1s and O1s high-resolution spectra of Sample 1000.

To further analyze the oxygen-containing functional surface groups, XPS measurements for the biomass charcoal samples were carried out. From the XPS survey spectra analyses, it can be found that the biomass charcoal samples have carbon and oxygen atoms (Fig. 5a), while their peaks have significant differences before and after 700 °C, that is, the O1s peaks gradually increase and the C1s peaks gradually decrease from Sample 300 to Sample 600. On the other hand, the O1s peaks gradually decrease and the C1s peaks gradually increase from Sample 700 to Sample 1000. To find a general change trend of the XPS survey spectra, a simple comparison of survey spectra (Fig. 5b) was conducted on Sample 300 (C of 72.66 at% and O of 23.56 at%), Sample 700 (C of 78.86 at% and O of 18.29 at%) and Sample 1000 (C of 82.18 at% and O of 15.22 at%), it could be found that the oxygen contents slowly decrease and the carbon contents slowly increase from low temperature to high temperature in the preparation process.

Figure 5c reveals that Sample 1000 has functional groups such as C=C/C–C (~ 284.7 eV), C–O (hydroxyl and epoxy, ~ 285.6 eV), C=O (carbonyl, ~ 287.1 eV), and O–C=O (carboxyl, ~ 289.7 eV)^30,31^. Figure 5d demonstrates that Sample 1000 has functional groups such as C=O (531.0 eV), O–C=O (532.0 eV), and C–O (533.0 eV)^30,31^. Therefore, the XPS measurements prove that the biomass charcoal samples have oxygen-containing functional surface groups, consistent with the above test results of FTIR and Raman spectra.

Based on the SEM, BET, FTIR, XRD, Raman spectra and XPS analyses mentioned above, it is reasoned that biomass charcoal from peanut shells can effectively form after 700 °C, and the structure of biomass charcoal can be gradually optimized from 700 to 1000 °C. On the other hand, there is no inert gas protection in the pyrolysis process and the subsequent process of natural cooling, which leads the biomass charcoal to contain a certain amount of oxygen-containing groups. Moreover, the hierarchical porous structure of biomass charcoal was derived from in-built template of the structure of peanut shell^12^. Therefore, the Sample 1000 should possess more excellent micromorphology with abundant oxygen-containing functional groups, which are helpful for the absorption of dangerous heavy metal ions and persistent organic pollutants from water.

### Adsorptive property

The biomass charcoal samples with large surface area, porous structure and abundant functional groups should be expected to work as excellent adsorbent to adsorb heavy metal ions and organic compounds from water. The potential adsorption application of the biomass charcoal samples could be verified as follows.

**Figure 6:**
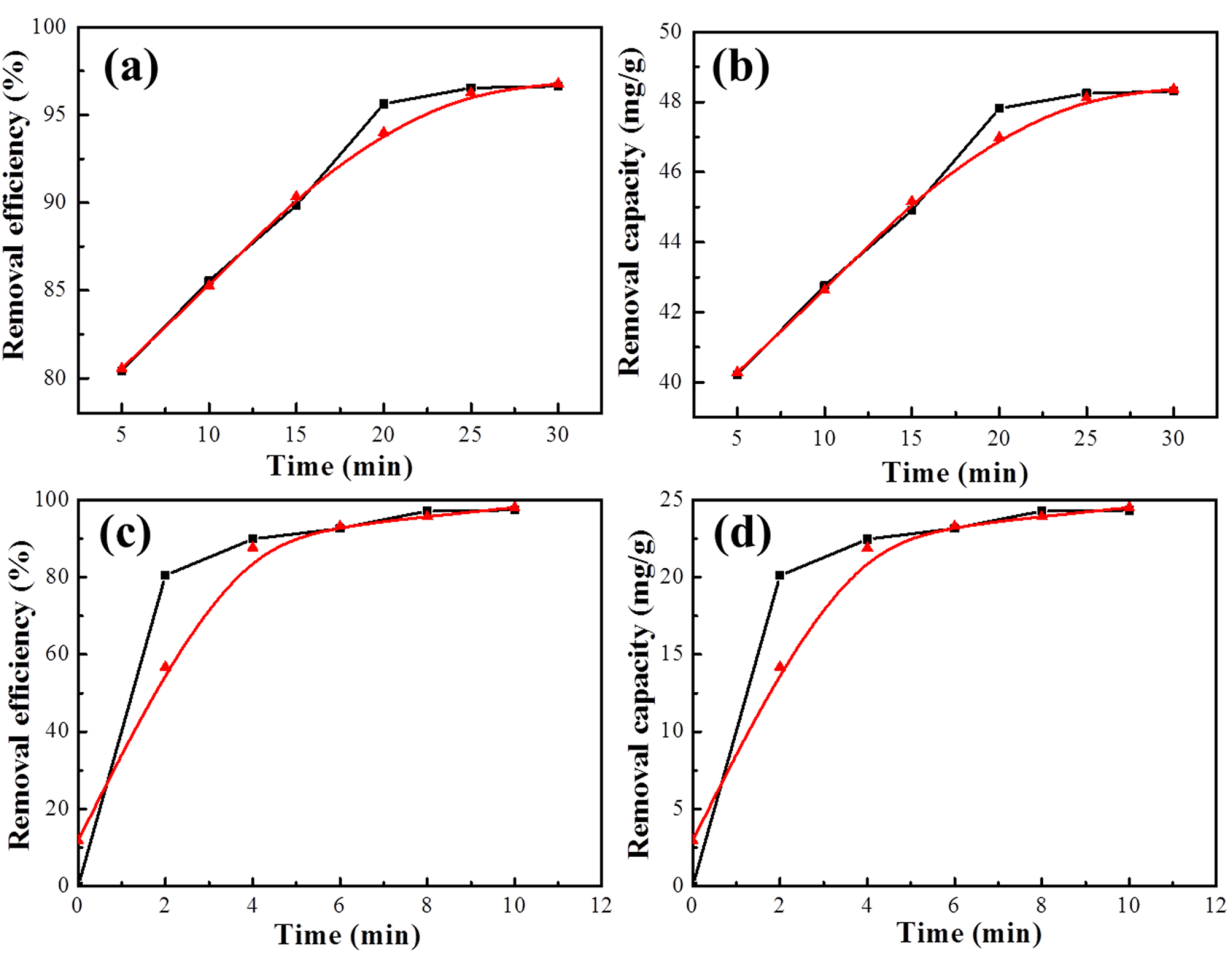
(**a**) Removal efficiency and (**b**) removal capacity of Sample 1000 in solution of Pb (II) (100 mg/L)**,** (**c**) removal efficiency and (**d**) removal capacity of Sample 1000 in solution of MB (50 mg/L).

The Sample 1000 was used as an adsorbent to remove Pb^2+^ from water. The adsorption equilibrium time of Pb^2+^ was around 25 min (Fig. 6a) with the removal efficiency of 96.5% and the equilibrium capacity (Q_e_) of 48 mg/g (Fig. 6b), suggesting that Sample 1000 can quickly remove Pb^2+^ from water with C_0_ = 100 mg/L of Pb^2+^ and 2 g/L of adsorbent. The adsorption capacity of Sample 1000 was higher than that of some reported in the literature^32–34^.

During the adsorbent of Sample 1000 was immerged in aqueous solution of MB (50 mg/L), the MB dye was quickly absorbed in only 2 min to reach 80.1% at room temperature. It can be seen that MB adsorption reaches equilibrium at around 8 min and the removal efficiency is 97.1% (Fig. 6c). The color of MB solution changed from dark-blue through pale and finally to colorless after a simple filtration, showing that MB can be quickly removed from water. The equilibrium capacity (Q_e_) could increase to 24 mg/g at around 8 min (Fig. 6d). This adsorbent could effectively adsorb MB and the adsorption capacity was higher than that of the TiO_2_ nanowire hierarchical membrane as previously reported^35^.

In our experiments, the waste peanut shells were ground to fine powders and utilized as raw materials to prepare biomass charcoal materials in an electric muffle furnace. Moreover, activating agents and inert gas protection were not needed in the preparation process. There are abundant sources of raw materials, the procedure and equipment is simple, thus the production cost is very low. Although some wastes have inevitably been produced in the preparation process, these wastes can also be recycled by an appropriate way. The obtained biomass charcoal samples with large surface area, hierarchical porous structure and abundant oxygen-containing functional groups would have many applications, such as adsorbent, catalyst support and filters.

## Conclusion

In this work, the biomass charcoal materials with large surface area, hierarchical porous structure and abundant oxygen-containing functional group were prepared through a simple and facile process without chemical activation and nitrogen protection. The obtained sample with excellent adsorptive property was used as effective functional adsorbent for the removal of Pb(II) and MB from waste water. Systematic investigations are underway to further probe the affecting factors and the formation mechanism of the biomass charcoal materials, and it will provide a beneficial reference in the recovery of biomass waste for many applications.
